# Design, synthesis and biological evaluation of benzoxazolinone-containing 1,3,4-thiadiazoles as TNF-α inhibitors

**DOI:** 10.1016/j.heliyon.2019.e01503

**Published:** 2019-04-22

**Authors:** Saqlain Haider, Mohammad Sarwar Alam, Hinna Hamid, Abhijeet Dhulap, Deepak Kumar

**Affiliations:** a Department of Chemistry, School of Chemical and Life Sciences, Jamia Hamdard, New Delhi, 110 062, India; b Unit for Research and Development of Information Products - CSIR, Pune, 411038, India; c Department of Pharmaceutical Chemistry, School of Pharmaceutical Sciences, Shoolini University, Solan, H.P., 173229, India

**Keywords:** Organic chemistry, Pharmaceutical chemistry

## Abstract

A library of nineteen benzoxazolinone-based 1,3,4-thiadiazoles has been synthesized and screened for their anti-inflammatory activity. The compound **1f** exhibited a potent anti-inflammatory activity with an inhibition of 65.83% and 32.50% after 3 h and 5 h respectively. It also exhibited a significant *in vitro* (p < 0.01), TNF- α inhibitory activity with 51.44 % inhibition. The compound **1f** showed hydrogen bonding with GLN 61 and interactions with TYR 119, TYR 151 and GLY 121. The histopathology report showed that none of the compounds caused gastric ulceration. The results from the *in vivo* & *in vitro* antiinflammatory activity along with *In Silico* studies exhibit that benzoxazolinone-based 1,3,4-thiadiazoles may be used in the future development of anti-inflammatory drugs.

## 1 Introduction

Gastric haemorrhage and ulceration are the two most serious side effects associated with Non-steroidal anti-inflammatory drugs (NSAID) available currently in the market [1]. Their consumption in a long run may cause gastric wounds [2]. Monocytes, macrophages, B-cells, T-cells and fibroblast-like synoviocytes are involved in the production of pro-inflammatory mediators like TNF-α [3]. The role of TNF-α in the inflammatory reactions like shock, tissue destruction and organ failure is already reported [4, 5, 6]. Inflammatory reactions are marked by the production of substantial amounts of proinflammatory mediators and they affect the immune system by supressing the production of T and B cells, as well as cytokine production. The intensity of a disease can be minimized by controlling the production of these molecules.

Five-membered heterocyclic rings are present in compounds with important chemical significance showing a broad array of biological activities. Amongst these five-membered heterocyclic compounds, 2,5-disubstituted 1,3,4-thiadiazoles have been found to exhibit potent medicinal properties with their –N=C-S- group being at the centre of focus. The better liposolubility of the thiadiazoles is due to the presence of sulfur atom which in turn enhances its mesoionic nature and the ability to cross the cellular membrane [7, 8]. The thiadiazoles cross the cellular membrane and interact with specific targets due to the fact that they possess a dense, polarizable neutral electronic charge. The toxicity of the thiadiazoles is still a challenge even though they have several advantages. Some thiadiazole containing drugs present in the market include cefazedone, timolol, acetazolamide, cefazolin sodium, and xanomeline. The antinociceptive and anti-inflammatory properties of thiadiazoles are well reported in the literature [9, 10, 11, 12]. On the other hand the compounds containing 2-benzoxazolinone scaffold exhibit a myriad of activities including antimicrobial [13, 14], antinociceptive [15], anti-inflammatory [16] and anti-HIV activities [17].

Considering the biological importance of benzoxazolinone and 1,3,4-thiadiazoles as anti-inflammatory and antinociceptive agents we have combined these two important scaffolds under one construct through a methylene linkage. The current work has been designed with an aim to develop new molecules with a potential to inhibit the increase in the level of proinflammatory mediator such as TNF-α without inducing any gastric ulceration. We report herein the design and synthesis of benzoxazolinone-containing 1,3,4-thiadiazoles and their *in vivo* anti-inflammatory and analgesic activities along with their *in vitro* TNF-α assay and their *in silico* studies. The active compounds have also been evaluated for their gastric risk evaluation.

## 2 Materials and methods

All the reagents used in this study were purchased from Merck (India), Sigma Aldrich and Spectrochem. All melting points were uncorrected and measured using Veego VMPDS apparatus, IR spectra were recorded as potassium bromide pellets on a Perkin Elmer 1650 spectrophotometer (USA), ^1^H NMR spectra was determined on a Bruker (400) spectrometer and chemical shifts are expressed as ppm against TMS as internal reference. Mass spectra were recorded on 70 eV (EI Ms-QP 1000EX, Shimadzu, Japan). Column chromatography was performed on silica gel (60–120 mesh). Elemental analysis was carried out using Elementar Vario EL III elemental analyser. Elemental analysis data is reported in % standard.

### 2.1 Chemistry

#### 2.1.1 Synthesis the compound 3-[5-(3-Chloro-phenylamino)-[1,3,4]thiadiazol-2-yl-methyl]-6-nitro-3H-benzooxazol-2- one(**1a**)

2-amino-5-nitrophenol (6.5 mmol, 1 g) and 1,1-carbonyldiimidazole (16 mmol, 2.52 g) were dissolved in dry THF (25 ml) and the solution was refluxed for 5 h. The reaction mixture was poured on ice and the yellow coloured precipitate thus obtained was dried and purified by column chromatography over silica gel using EtOAc: Hexane (30:70), to obtain pure 6-nitrobenzo [*d*]oxazol-2(3*H*)-one. A mixture of pure 6-nitrobenzo [*d*]oxazol-2(3*H*)-one (5.5 mmol, 1 g), ethylbromoacetate (5.5 mmol, 928 mg) and K_2_CO_3_ (16 mmol, 2.30 g) was refluxed in dry acetone for 8 h. The reaction mixture was then poured on crushed ice and the yellow precipitate thus obtained was filtered, dried and purified by column chromatography over silica gel using EtOAc: Hexane (20:80) to obtain a pure ester derivative of 6-nitrobenzo [*d*]oxazol-2(3*H*)-one. The ester derivative, ethyl-2-(6-nitro-2-oxobenzo [*d*]oxazol-3(2*H*)-yl)acetate (3 mmol, 800 mg) and hydrazine hydrate (3.6 mmol, 112 μl) were dissolved in absolute ethanol and the solution was refluxed for 3 h to obtain an orange coloured 2-(6-nitro-2-oxobenzo [*d*]oxazol-3(2*H*)-yl)acetohydrazide which was purified by crystallisation in hot ethanol. The pure acetohydrazide (3.9 mmol, 1 g) was reacted with 1-chloro-3-isothiocyanatobenzene (3.9 mmol, 672 mg) to obtain the thiosemicarbazide, *N*-(3-chlorophenyl)-2-(2-(6-nitro-2-oxobenzo [*d*]oxazol-3(2*H*)-yl)acetyl)hydrazine-1-carbothioamide as a yellow solid which was purified by crystallisation in ethanol. The thiosemicarbazide was dissolved in H_2_SO_4_ at 0 °C, stirred for an hour and then neutralised with aqueous ammonia solution to give a crude product which was further purified by crystallisation in ethanol to give pure (3-[5-(3-chlorophenylamino)-[1,3,4]thiadiazol-2-ylmethyl]-6-nitro-3H-benzooxazol-2-one in 74% yield as the final compound.

#### 2.1.2 3-[5-(3-Chlorophenylamino)-[1,3,4]thiadiazol-2-yl-methyl]-6-nitro-3H-benzooxazol-2- one(**1a**)

Green powder; Yield 74%; m.p. 232–233 °C; IR (KBr) ν (cm^−1^) 3367, 3329, 1808, 1622, 1596; ^1^H NMR (DMSO-d_6_, 300 MHz): δ 5.50 (s, 2H), 7.04 (d, 1H, *J* = 6.6 Hz), 7.34–7.36 (m, 2H), 7.57 (d, 1H, *J* = 8.7 Hz), 7.87 (s, 1H), 8.27 (dd, 1H, *J* = 2.1, 8.4 Hz), 8.34 (d, 1H, *J* = 2.1 Hz), 10.55 (s, 1H); ^13^C NMR (DMSO-d_6_, 75 MHz): δ 111.27, 114.69, 121.13, 122.08, 126.31, 126.84, 135.88, 138.70, 141.73, 146.73, 146.82, 148.00, 158.37, 158.60, 170.41, ‘One of the ^13^C signals is missing due to accidental signal equivalence’; MS (ESI) m/z: 404 (M+1)^+^; Anal. Calcd. for C_16_H_10_ClN_5_O_4_S: C, 47.59; H, 2.50; N, 17.34; S, 7.94%. Found C, 47.56; H, 2.48; N, 17.35; S, 7.92%.

#### 2.1.3 3-[5-(4-Methoxyphenylamino)-[1,3,4]thiadiazol-2-yl-methyl]-6-nitro-3H-benzooxazol-2-one(**1b**)

Green powder; Yield 71%; m.p. 214–215 °C; IR (KBr) ν (cm^−1^) 3319, 3300, 1778, 1604, 1545, 1253; ^1^H NMR (DMSO-d_6_, 300 MHz): δ 3.71 (s, 3H), 5.45 (s, 2H), 6.91 (d, 2H, *J* = 7.2 Hz), 7.46 (d, 1H, *J* = 8.7 Hz), 7.55 (d, 2H, *J* = 8.4 Hz), 8.25–8.34 (m, 2H), 10.32 (s, 1H); ^13^C NMR (DMSO-d_6_, 75 MHz): δ 54.32, 114.22, 114.34, 122.21, 123.11, 125.22, 125.52, 134.34, 136.73, 140.72, 145.25, 148.34, 156.23, 158.54, 171.23; MS (ESI) m/z: 400 (M+1)^+^; Anal. Calcd. for C_17_H_13_N_5_O_5_S: C, 51.12; H, 3.28; N, 17.54; S, 8.03%. Found C, 51.13; H, 3.29; N, 17.55; S, 8.04%.

#### 2.1.4 3-[5-(4-Fluorophenylamino)-[1,3,4]thiadiazol-2-yl-methyl]-6-nitro-3H-benzooxazol-2-one(**1c**)

Yellowish green powder; Yield 75%; m.p. 209–210 °C; IR (KBr) ν (cm^−1^) 3398, 1764, 1623, 1349, 1232, 1093; ^1^H NMR (DMSO-d_6_, 300 MHz): δ 5.47 (s, 2H), 7.17 (t, 2H, *J* = 8.7 Hz), 7.54–7.61 (m, 3H), 8.26 (dd, 1H, *J* = 8.7, 2.1 Hz), 8.33 (d, 1H, *J* = 1.8 Hz), 10.43 (s, 1H); ^13^C NMR (DMSO-d_6_, 75 MHz): δ 114.32, 114.43, 121.23, 123.34, 126.43, 126.52, 136.63, 137.32, 145.21, 147.31, 147.62, 163.7, 167.6 (C, C–F, J = 240 Hz), 170.41; MS (ESI) m/z: 388 (M+1)^+^; Anal. Calcd. for C_16_H_10_FN_5_O_4_S: C, 49.61; H, 2.60; N, 18.08; S, 8.28%. Found C, 49.59; H, 2.58; N, 18.07; S, 8.27%.

#### 2.1.5 3-[5-(4-Bromophenylamino)-[1,3,4]thiadiazol-2-yl-methyl]-6-nitro-3H-benzooxazol-2-one(**1d**)

White powder; Yield 80%; m.p. 220–221 °C; IR (KBr) ν (cm^−1^) 3397, 3367, 1808, 1622, 1340, 1237; ^1^H NMR (DMSO-d_6_, 300 MHz): δ 5.48 (s, 2H), 7.46–7.57 (m, 5H), 8.24–8.31 (m, 2H), 10.54 (s, 1H); ^13^C NMR (DMSO-d_6_, 75 MHz): δ 106.55, 109.96, 113.84, 119.86, 121.59, 132.27, 137.01, 140.11, 141.99, 143.24, 153.37, 153.86, 165.72, ‘One of the ^13^C signals is missing due to accidental signal equivalence’; MS (ESI) m/z: 448 (M+2)^+^; Anal. Calcd. for C_16_H_10_BrN_5_O_4_S: C, 42.87; H, 2.25; N, 15.62; S, 7.15%. Found C, 42.86; H, 2.26; N, 15.61; S, 7.16%.

#### 2.1.6 6-Nitro-3-(5-phenylamino-[1,3,4]thiadiazol-2-yl-methyl)-3H-benzooxazol-2-one(**1e**)

Yellow powder; Yield 68%; m.p. 195–196 °C; IR (KBr) ν (cm^−1^) 3669, 1918, 1784, 1622; ^1^H NMR (DMSO-d_6_, 300 MHz): δ 5.47 (s, 2H), 6.99 (t, 1H, *J* = 7.5 Hz), 7.32 (t, 2H, *J* = 7.8 Hz), 7.55 (d, 3H, *J* = 8.4 Hz), 8.24–8.31 (m, 2H), 10.37 (s, 1H); ^13^C NMR (DMSO-d_6_, 75 MHz): δ 106.54, 109.94, 117.93, 121.58, 122.58, 129.56, 137.00, 140.84, 141.9, 143.24, 152.79, 153.86, 166.13, ‘One of the 13C signals is missing due to accidental signal equivalence’; MS (ESI) m/z: 370 (M+1)^+^; Anal. Calcd. for C_16_H_11_N_5_O_4_S: C, 52.03; H, 3.00; N, 18.96; S, 8.68%. Found C, 52.03; H, 3.00; N, 18.96; S, 8.68%.

#### 2.1.7 3-[5-(4-Methoxyphenylamino)-[1,3,4]thiadiazol-2-yl-methyl]-6-methyl-3H-benzooxazol-2-one(**1f**)

White powder; Yield 78%; m.p. 186–187 °C; IR (KBr) ν (cm^−1^) 3418, 1775, 1599, 1494, 1255; ^1^H NMR (DMSO-d_6_, 300 MHz): δ 2.33 (s, 3H), 3.71 (s, 3H), 5.31 (s, 2H), 6.91–7.22 (m, 5H), 7.46 (d, 1H, *J* = 6.0 Hz), 7.74 (s, 1H), 10.24 (s, 1H); ^13^C NMR (DMSO-d_6_, 75 MHz): δ 21.32, 52.24, 115.23, 116.27, 123.43, 124.56, 126.45, 127.38, 133.85, 135.37, 141.32, 142.36, 145.54, 154.23, 162.43; MS (ESI) m/z: 369 (M+1)^+^; Anal. Calcd. for C_18_H_16_N_4_O_3_S: C, 58.68; H, 4.38; N, 15.21; S, 8.70%. Found C, 58.66; H, 4.36; N, 15.20; S, 8.69%.

#### 2.1.8 3-[5-(4-Chlorophenylamino)-[1,3,4]thiadiazol-2-yl-methyl]-6-methyl-3H-benzooxazol-2-one(**1g**)

White powder; Yield 83%; m.p. 190–191 °C; IR (KBr) ν (cm^−1^) 3228, 3182, 1757, 1543, 1423; ^1^H NMR (DMSO-d_6_, 300 MHz): δ 2.34 (s, 3H), 5.35 (s, 2H), 7.05 (d, 1H, *J* = 8.1 Hz), 7.17–7.22 (m, 2H), 7.38 (d, 2H, *J* = 9.0 Hz), 7.61 (d, 2H, *J* = 8.7 Hz), 10.19 (s, 1H); ^13^C NMR (DMSO-d_6_, 75 MHz): δ 20.80, 58.03, 109.10, 110.33, 118.96, 124.34, 127.77, 128.84, 132.58, 139.13, 142.00, 153.61, 165.07, ‘Two of the ^13^C signals are missing due to accidental signal equivalence’; MS (ESI) m/z: 373 (M+1)^+^; Anal. Calcd. for C_17_H_13_ClN_4_O_2_S: C, 54.77; H, 3.51; N, 15.03; S, 8.60%. Found C, 54.75; H, 3.50; N, 15.02; S, 8.59%.

#### 2.1.9 3-[5-(4-Ethoxyphenylamino)-[1,3,4]thiadiazol-2-yl-methyl]-6-methyl-3H-benzooxazol-2-one(**1h**)

White powder; Yield 65%; m.p. 178–179 °C; IR (KBr) ν (cm^−1^) 3356, 1775, 1611, 1349, 1263, 1140; ^1^H NMR (DMSO-d_6_, 300 MHz): δ 1.30 (t, 3H, *J* = 6.6 Hz), 2.33 (s, 3H), 3.96 (q, 2H, 6.9 Hz), 5.30 (s, 2H), 6.89 (d, 2H, *J* = 8.7 Hz), 7.04 (d, 1H, *J* = 7.8 Hz), 7.16–7.22 (m, 2H), 7.44 (d, 2H, *J* = 8.7 Hz), 10.27 (s, 1H); ^13^C NMR (DMSO-d_6_, 75 MHz): δ 15.12, 21.32, 63.62, 109.63, 110.83, 115.28, 119.86, 124.84, 128.31, 133.03, 134.12, 142.50, 152.82, 154.00, 154.42, ‘Two of the ^13^C signals are missing due to accidental signal equivalence’; MS (ESI) m/z: 383 (M+1)^+^; Anal. Calcd. for C_19_H_18_N_4_O_3_S: C, 59.67; H, 4.74; N, 14.65; S, 8.38%. Found C, 59.66; H, 4.72; N, 14.63; S, 8.37%.

#### 2.1.10 3-[5-(4-Fluorophenylamino)-[1,3,4]thiadiazol-2-yl-methyl]-6-methyl-3H-benzooxazol-2-one(**1i**)

White powder; Yield 71%; m.p. 202–203 °C; IR (KBr) ν (cm^−1^) 3828, 1755, 1623, 1571, 1389, 1263, 1221; ^1^H NMR (DMSO-d_6_, 300 MHz): δ 2.33 (s, 3H), 5.34 (s, 2H), 7.04 (d, 1H, *J* = 8.1Hz), 7.14–7.22 (m, 4H), 7.57–7.61 (m, 2H), 10.32 (s, 1H); ^13^C NMR (DMSO-d_6_, 75 MHz): 21.32, 109.64, 110.85, 115.94, 116.24, 119.71, 119.81, 124.86, 128.32, 133.07, 137.29, 142.54, 149.42, 160.6, 164.5 (C, C–F, J = 240 Hz); MS (ESI) m/z: 357 (M+1)^+^; Anal. Calcd. for C_17_H_13_FN_4_O_2_S: C, 57.29; H, 3.68; N, 15.72; S, 9.00%. Found C, 57.27; H, 3.67; N, 15.71; S, 8.98%.

#### 2.1.11 3-[5-(3-Chlorophenylamino)-[1,3,4]thiadiazol-2-yl-methyl]-6-methyl-3H-benzooxazol-2-one(**1j**)

White powder; Yield 65%; m.p. 180–181 °C; IR (KBr) ν (cm^−1^) 3134, 3099, 1750, 1603, 1309, 1263, 1175; ^1^H NMR (DMSO-d_6_, 300 MHz): δ 2.33 (s, 3H), 5.36 (s, 2H), 7.04 (d, 2H, *J* = 8.1Hz), 7.17–7.23 (m, 2H), 7.31–7.36 (m, 2H), 7.86 (s, 1H), 10.26 (s, 1H); ^13^C NMR (DMSO-d_6_, 75 MHz): δ 21.35, 109.70, 110.84, 116.37, 117.31, 122.05, 124.82, 128.42, 131.14, 132.97, 133.94, 142.09, 142.56, 154.01, 154.41, 165.42, ‘One of the ^13^C signals is missing due to accidental signal equivalence’; MS (ESI) m/z: 372 (M)^+^, 371 (M-1)^+^; Anal. Calcd. for C_17_H_13_ClN_4_O_2_S: C, 54.77; H, 3.51; N, 15.03; S, 8.60%. Found C, 54.75; H, 3.49; N, 15.01; S, 8.59%.

#### 2.1.12 6-Methyl-3-(5-phenylamino-[1,3,4]thiadiazol-2-yl-methyl)-3H-benzooxazol-2-one(**1k**)

Green powder; Yield 83%; m.p. 193–194 °C; IR (KBr) ν (cm^−1^) 3150, 3109, 1756, 1610, 1563, 1368, 1174; ^1^H NMR (DMSO-d_6_, 300 MHz): δ 2.33 (s, 3H), 5.34 (s, 2H), 6.97–7.03 (m, 2H)), 7.17–7.23 (m, 2H), 7.30–7.35 (m, 2H), 7.56 (d, 2H, *J* = 7.8 Hz), 10.41 (s, 1H); ^13^C NMR (DMSO-d_6_, 75 MHz): δ 21.35, 109.68, 110.84, 117.94, 122.55, 124.81, 128.40, 129.55, 132.96, 140.85, 142.55, 153.58, 154.00, 165.90, ‘One of the 13C signals is missing due to accidental signal equivalence’; MS (ESI) m/z: 339 (M+1)^+^; Anal. Calcd. for C_17_H_14_N_4_O_2_S: C, 60.34; H, 4.17; N, 16.56; S, 9.48%. Found C, 60.32; H, 4.16; N, 16.57; S, 9.47%.

#### 2.1.13 3-[5-(4-Fluorophenylamino)-[1,3,4]thiadiazol-2-yl-methyl]-5-methyl-3H-benzooxazol-2-one(**1l**)

White powder; Yield 70%; m.p. 189–190 °C; IR (KBr) ν (cm^−1^) 3163, 3096, 1754, 1622, 1537, 1386, 1357; ^1^H NMR (DMSO-d_6_, 300 MHz): δ 2.33 (s, 3H), 5.33 (s, 2H), 6.96 (d, 1H, *J* = 8.1 Hz), 7.15–7.27 (m, 4H), 7.57–7.62 (m, 2H), 10.43 (s, 1H); ^13^C NMR (DMSO-d_6_, 75 MHz): δ 22.34, 105.23, 108.32, 112.37, 113.86, 121.43, 134.31, 136.21, 142.22, 143.47, 145.44, 152.43, 162.6, 166.5 (C, C–F, J = 240 Hz), 166.21; MS (ESI) m/z: 357 (M+1)^+^; Anal. Calcd. for C_17_H_13_FN_4_O_2_S: C, 57.29; H, 3.68; N, 15.72; S, 9.00%. Found C, 57.28; H, 3.67; N, 15.73; S, 9.02%.

#### 2.1.14 3-[5-(4-Bromophenylamino)-[1,3,4]thiadiazol-2-yl-methyl]-5-methyl-3H-benzooxazol-2-one(**1m**)

White powder; Yield 80%; m.p. 176–177 °C; IR (KBr) ν (cm^−1^) 3398, 1764, 1623, 1545, 1349, 1232, 1093; ^1^H NMR (DMSO-d_6_, 300 MHz): δ 2.33 (s, 3H), 5.35 (s, 2H), 6.96–7.02 (m, 3H), 7.48–7.62 (m, 4H), 10.30 (s, 1H); ^13^C NMR (DMSO-d_6_, 75 MHz): δ 20.59, 108.83, 111.77, 113.78, 119.88, 130.69, 132.26, 132.61, 139.43, 140.14, 154.17, 154.33, 165.48, ‘Two of the 13C signals are missing due to accidental signal equivalence’; MS (ESI) m/z: 417 (M+2)^+^; Anal. Calcd. for C_17_H_13_BrN_4_O_2_S: C, 48.93; H, 3.14; N, 13.43; S, 7.68%. Found C, 48.92; H, 3.15; N, 13.42; S, 7.66%.

#### 2.1.15 3-[5-(4-Bromophenylamino)-[1,3,4]thiadiazol-2-yl-methyl]-3H-benzooxazol-2-one(**1n**)

White powder; Yield 77%; m.p. 182–183 °C; IR (KBr) ν (cm^−1^) 3218, 1796, 1616, 1436, 1348, 1241, 1155; ^1^H NMR (DMSO-d_6_, 300 MHz): δ 5.39 (s, 2H), 7.17–7.24 (m, 2H), 7.32 (d, 1H, *J* = 6.3 Hz), 7.39 (d, 1H, *J* = 7.5 Hz), 7.50 (d, 2H, *J* = 8.7 Hz), 7.56 (d, 2H, *J* = 8.7 Hz), 10.56 (s, 1H); ^13^C NMR (DMSO-d_6_, 75 MHz): δ 114.83, 115.11, 118.57, 124.62, 127.98, 129.30, 135.55, 136.99, 144.87, 147.22, 158.64, 158.79, 170.34, ‘One of the ^13^C signals is missing due to accidental signal equivalence’; MS (ESI) m/z: 403 (M+2)^+^, 405 (M+3)^+^; Anal. Calcd. for C_16_H_11_BrN_4_O_2_S: C, 47.66; H, 2.75; N, 13.89; S, 7.95%. Found C, 47.65; H, 2.73; N, 13.91; S, 7.97%.

#### 2.1.16 3-[5-(4-Chlorophenylamino)-[1,3,4]thiadiazol-2-yl-methyl]-3H-benzooxazol-2-one(**1o**)

White powder; Yield 70%; m.p. 173–174 °C; IR (KBr) ν (cm^−1^) 3267, 3196, 1753, 1541, 1487, 1403, 1090; ^1^H NMR (DMSO-d_6_, 300 MHz): δ 5.39 (s, 2H), 7.17–7.27 (m, 2H), 7.31–7.39 (m, 3H), 7.50 (d, 1H, *J* = 6.0 Hz), 7.61 (d, 2H, *J* = 8.4 Hz), 10.27 (s, 1H); ^13^C NMR (DMSO-d_6_, 75 MHz): δ 106.32, 108.23, 114.85, 115.32, 119.72, 121.16, 132.49, 133.63, 137.23, 140.54, 153.28, 155.43, 162.41, ‘One of the ^13^C signals is missing due to accidental signal equivalence’; MS (ESI) m/z: 359 (M+1)^+^; Anal. Calcd. for C_16_H_11_ClN_4_O_2_S: C, 53.56; H, 3.09; N, 15.61; S, 8.94%. Found C, 53.54; H, 3.08; N, 15.59; S, 8.92%.

#### 2.1.17 3-[5-(4-Fluorophenylamino)-[1,3,4] thiadiazol-2-yl-methyl]-3H-benzooxazol-2-one(**1p**)

White powder; Yield 67%; m.p. 168–169 °C; IR (KBr) ν (cm^−1^)3316, 1797, 1615, 1386, 1302, 1081; ^1^H NMR (DMSO-d_6_, 300 MHz): δ 5.36 (s, 2H), 7.14 (d, 2H, *J* = 9.0 Hz), 7.25 (d, 2H, *J* = 8.1 Hz), 7.49 (d, 1H, *J* = 6.0 Hz), 7.53–7.76 (m, 3H), 10.30 (s, 1H); ^13^C NMR (DMSO-d_6_, 75 MHz): δ 107.82, 109.15, 115.91, 116.21, 119.67, 122.06, 131.09, 137.37, 141.64, 143.83, 154.13, 159.37, 160.5, 164.4 (C, C–F, J = 240 Hz); MS (ESI) m/z: 343 (M+1)^+^; Anal. Calcd. for C_16_H_11_FN_4_O_2_S: C, 56.13; H, 3.24; N, 16.37; S, 9.37%. Found C, 56.12; H, 3.23; N, 16.38; S, 9.38%.

#### 2.1.18 3-[5-(4-Nitrophenylamino)-[1,3,4]thiadiazol-2-yl-methyl]-3H-benzooxazol-2-one(**1q**)

Green powder; Yield 70%; m.p. 188–189 °C; IR (KBr) ν (cm^−1^) 3123, 1754, 1632, 1338, 1247, 1152; ^1^H NMR (DMSO-d_6_, 300 MHz): δ 5.44 (s, 2H), 7.27 (d, 2H, *J* = 8.1 Hz), 7.52 (d, 2H, *J* = 9.3 Hz), 7.80 (d, 2H, *J* = 9.0 Hz), 8.24 (d, 2H, *J* = 9.1 Hz), 10.29 (s, 1H); ^13^C NMR (DMSO-d_6_, 75 MHz): δ 106.55, 109.98, 116.38, 117.32, 121.59, 122.08, 131.16, 133.96, 137.03, 141.99, 142.08, 143.25, 153.66, 153.87; MS (ESI) m/z: 370 (M+1)^+^; Anal. Calcd. for C_16_H_11_N_5_O_4_S: C, 52.03; H, 3.00; N, 18.96; S, 8.68%. Found C, 52.02; H, 3.02; N, 18.98; S, 8.69%.

#### 2.1.19 5-Chloro-3-[5-(4-chlorophenylamino)-[1,3,4]thiadiazol-2-yl-methyl]-3H-benzooxazol-2-one(**1r**)

White powder; Yield 83%; m.p. 210–211 °C; IR (KBr) ν (cm^−1^) 3347, 3117, 1773, 1619, 1568, 1443, 1243, 1117; ^1^H NMR (DMSO-d_6_, 300 MHz): δ 5.40 (s, 2H), 7.21 (d, 1H, *J* = 8.1 Hz), 7.42 (d, 1H, *J* = 2.1 Hz), 7.37 (d, 1H, J = 9.0 Hz), 7.43 (d, 2H, *J* = 8.4 Hz), 7.61 (d, 2H, *J* = 9.0 Hz), 10.55 (s, 1H); ^13^C NMR (DMSO-d_6_, 75 MHz): δ 110.44, 111.78, 119.46, 122.86, 128.64, 129.39, 132.29, 141.25, 153.70, 153.83, 165.46, ‘Three of the 13C signals are missing due to accidental signal equivalence’; MS (ESI) m/z: 392 (M+1)^+^, 393 (M+2)^+^; Anal. Calcd. for C_16_H_10_Cl_2_N_4_O_2_S: C, 48.87; H, 2.56; N, 14.25; S, 8.15%. Found C, 48.86; H, 2.57; N, 14.26; S, 8.14%.

#### 2.1.20 5-Chloro-3-[5-(4-fluorophenylamino)-[1,3,4]thiadiazol-2-yl-methyl]-3H-benzooxazol-2-one(**1s**)

White powder; Yield 80%; m.p. 215–216 °C; IR (KBr) ν (cm^−1^) 3239, 3155, 1796, 1616, 1376, 1241, 1160; ^1^H NMR (DMSO-d_6_, 300 MHz): δ 5.38 (s, 2H), 7.15 (d, 1H, *J* = 9.0 Hz), 7.20 (d, 1H, *J* = 8.1 Hz), 7.23 (d, 1H, *J* = 8.1 Hz), 7.42 (d, 2H, *J* = 8.7 Hz), 7.52 (d, 2H, *J* = 2.1 Hz), 10.33 (s, 1H); ^13^C NMR (DMSO-d_6_, 75 MHz): δ 110.41, 111.75, 115.93, 116.23, 119.64, 119.74, 122.85, 128.64, 137.36, 141.24, 153.21, 153.82, 156.22, 159.39; MS (ESI) m/z: 376 (M)^+^, 378 (M+2)^+^; Anal. Calcd. for C_16_H_10_ClFN_4_O_2_S: C, 51.00; H, 2.68; N, 14.87; S, 8.51%. Found C, 51.02; H, 3.69; N, 14.86; S, 8.49%.

### 2.2 Pharmacology

#### 2.2.1 Animals

Central Animal House, Jamia Hamdard, New Delhi provided the Albino Wistar rats of either sex (150–200 g) which were used for the *in vivo* studies. The rats were safely kept in the rat cages at room temperature and served with water ad libitum. The *in vivo* experiments were done explicitly under the guidelines of the Institutional Animals Ethics Committee (registration number 173-Committee for the Purpose of Control and Supervision of Experiments on Animals, CPCSEA).

#### 2.2.2 Chemicals

The chemicals including potassium bromide, amino phenols, hydrazine hydrate, potassium carbonate, carrageenan, carboxymethylcellulose and indomethacin etc. were procured from Sigma Aldrich Chemicals Pvt. Limited, Bangalore, India.

#### 2.2.3 Anti-inflammatory activity

Carrageenan-induced hind paw edema method has been used for the evaluation of *in vivo* anti-inflammatory activity of all the synthesized compounds [18]. This method involves the subcutaneous injection of 0.1 ml of 1% freshly prepared saline solution of carrageenan into the right hind paw of rats resulting in the paw edema in rats. Indomethacin (0.05 mmol/kg) was administered orally to the rats as a positive control. The control group animals were orally given 0.9% of 0.1 ml of saline solution only. The test group animals were given an equimolar dosage of the synthesized compounds and the standard drug, Indomethacin, an hour prior to the administration of carrageenan. Plethysmometer was used to record the paw volumes after an interval of 3 h and 5 h for all the animals [19, 20].

#### 2.2.4 TNF-α assay

RPMI 1640 having 1 M HEPES, 100 U/mL penicillin, 10% FBS, and 2 mM glutamine was used to grow the macrophage cells. 100 mg/mL streptomycin was obtained by passage through a stainless mesh [20]. ELISA kits from e-Biosciences was used to measure the pro-inflammatory cytokine levels in the supernatants of cell culture collected from macrophage cells seeded with variable concentrations of compounds. Cytochalasin D (2.5 mM/1 × 10^6^ cells) and the macrophages were pre-incubated for 30 min prior to the start of the experiment and continued till the end. The cytokine levels were measured according to the protocols from the manufacturer.

Softmax program was used to study the results.

### 2.3 Antinociceptive activity

#### 2.3.1 Writhing test

The method reported by Koster was used to perform the writhing test in mice [21]. An intraperitoneal injection of 0.6% acetic acid (v/v) (80 mg/kg) was used to induce the writhes in the mice. Indomethacin at a dosage of 0.05 mmol/kg of body weight was administered orally to the mice. The test compounds were given orally at an equimolar dosage to groups of six animals each, 30 min before chemical inducement. After the injection of acetic acid, the number of muscular contractions was counted over a span of 20 min. The data is expressed as writhing numbers which represents the total number of writhes observed during 20 min.

#### 2.3.2 Tail immersion method

Tail immersion method is another method to study and analyze antinociception [22]. This method involves the screening of the rats for sensitivity by immersing their tails gently in hot water maintained at 55 °C [23]. Only those animals were selected for further studies which flicked their tails from the hot water in 5 s. This was done to avoid any thermal injury. 0.9% of 0.1mL of saline solution was administered orally to the control group. The test compounds and the standard drug (indomethacin) were given orally at a dosage of 0.05 mmol/kg of body weight.

The reaction time was measured at an interval of 30, 60 and 120 min after the administration of the test compounds. The reaction time prior to the drug administration was called as the basal reaction time.

#### 2.3.3 Ulcerogenic activity

The test compounds showing significant anti-inflammatory & antinociceptive activities comparable with indomethacin were finally screened for their ulcerogenic risk evaluation. The ulcerogenic risk evaluation was carried out at three times higher dosage in comparison to that used for the *in vivo* anti-inflammatory activity, i.e. 0.15 mmol/kg body weight of the test compounds and indomethacin was used for the ulcerogenic study. The animals were sacrificed at the end of the study. The gastric tolerance of the test compounds was found to be better than that of indomethacin because none of them caused any disruption of gastric epithelial cells or gastric ulceration at the used oral dosages [24].

#### 2.3.4 Molecular docking studies on TNF-α

The molecular docking study against the TNF-α protein was done according to a previously reported procedure [25]. In short, the protein data bank [PDB] was used to obtain the crystallized structure of 2AZ5 which was used as the target docking. 2AZ5 structure from PDB is reported with celecoxib which inhibits it. The Protein Preparation Wizard (PPZ) was used to import the structure of 2AZ5 into the Schrodinger. The prime interface was used to add the missing hydrogens and the other atoms. The water molecules which were not needed were removed. This was followed by the optimization of the protein to give the least energetic and structurally correct target protein. Since the target protein already had the site for reference ligand therefore the grid was automatically generated by selecting the ligand as the reference ligand. The grid was finally validated and used for docking the new ligands to predict their docking score. Maestro was used to draw the chemical structures and LigPrep module was used to geometrically refine them. 3-D structures were generated from the 2-D structures in this module and subjected to the OPLS-2005 force field to generate single low energy 3-D structure for each input. Glide software with extra precision and XP descriptor was used to do the docking. The generated ligand poses were further screened through the filters to examine spatial fit of the ligand into the active site. The ligand poses which passed the initial screening were subjected to further evaluation.

## 3 Results and discussion

### 3.1 Chemistry

**Fig. 1:**
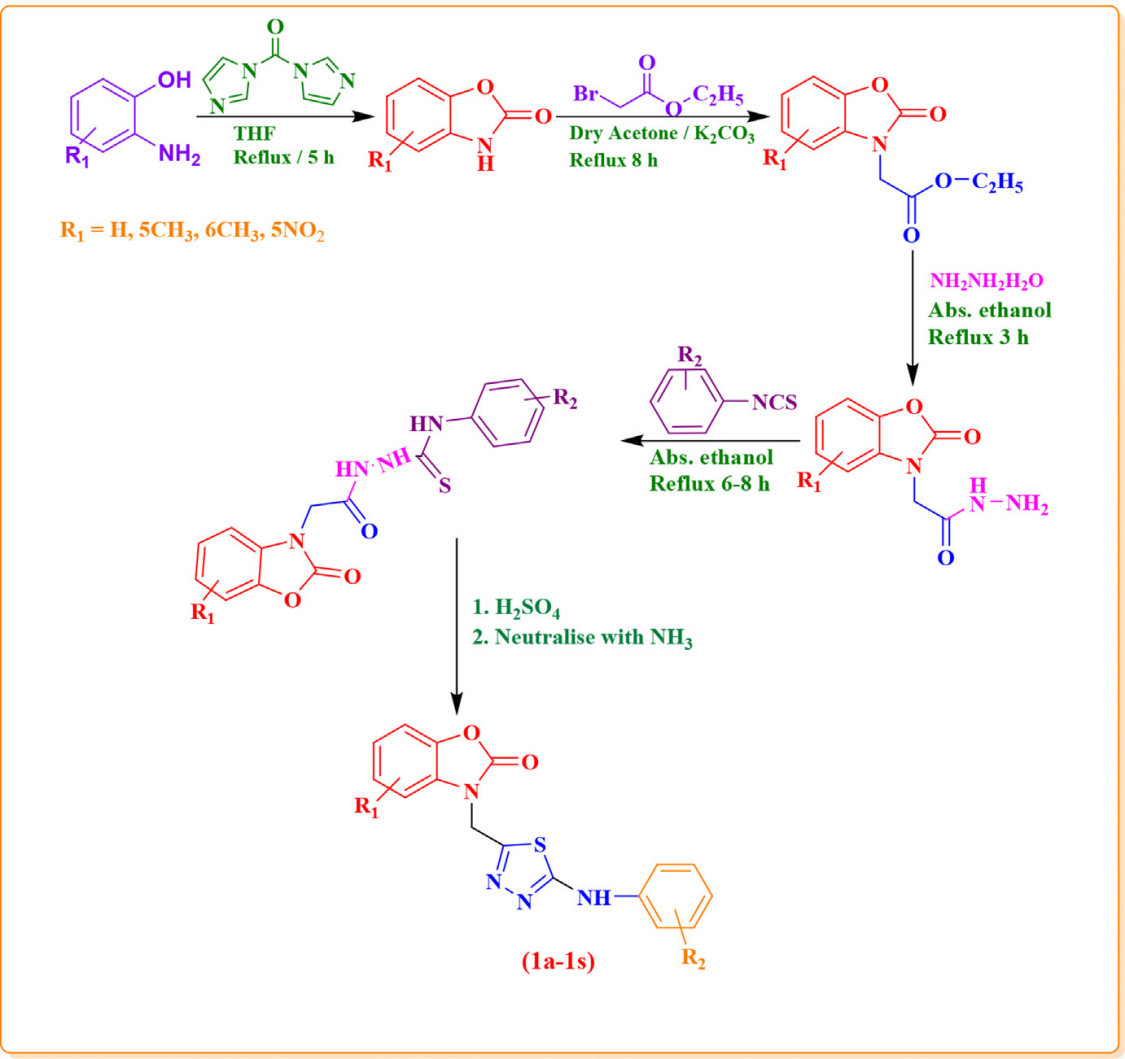
Synthesis of benzoxazolinone-based 1,3,4-thiadiazoles.

**Fig. 2:**
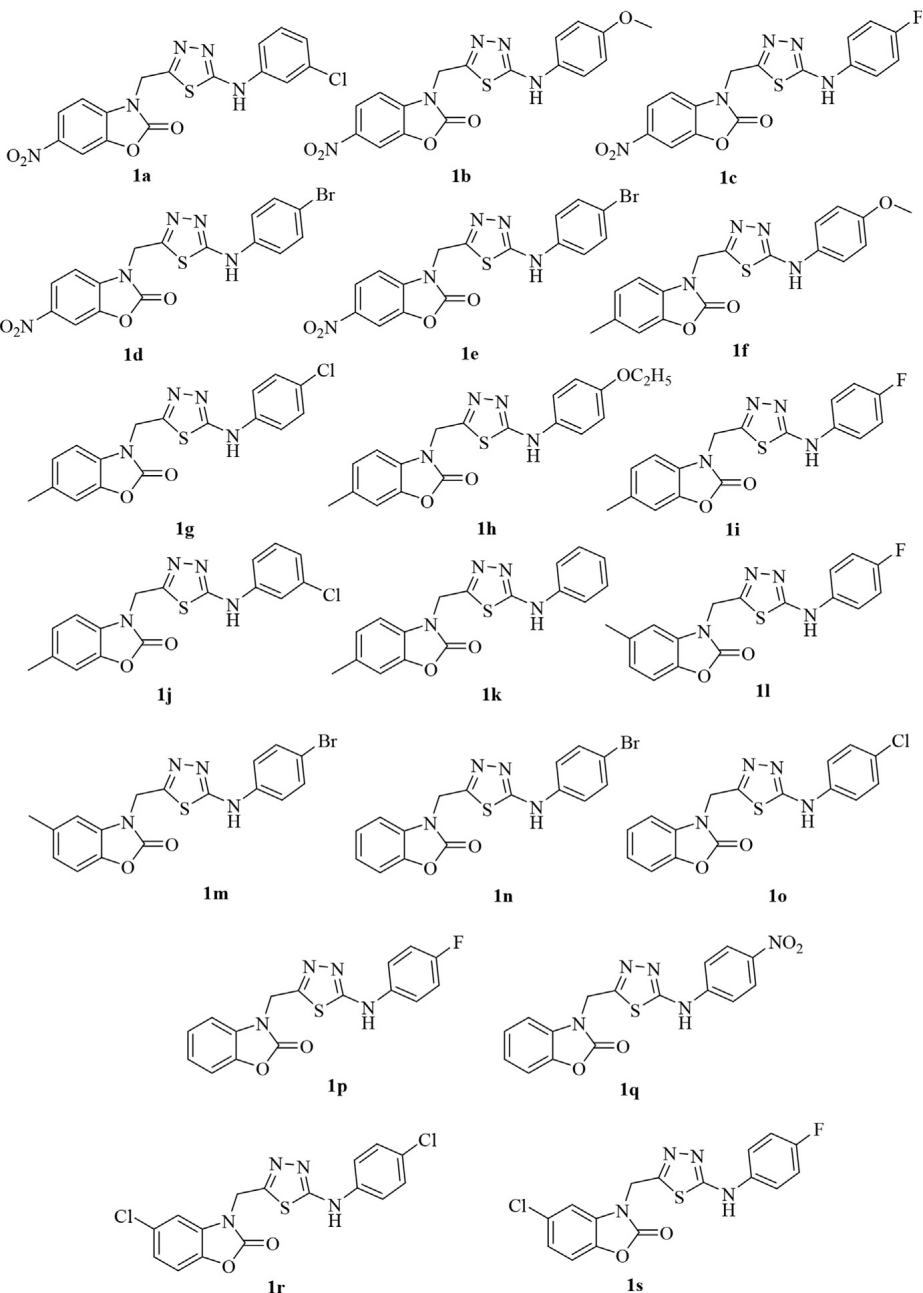
Structures of the synthesized compounds.

A focused library of eighteen novel benzoxazolinone-based 1,3,4-thiadiazoles (**1a–1s)** has been synthesized, starting from different *o*-aminophenol derivatives. As shown in Fig. 1, 2-aminophenol derivatives were refluxed with 1,1-carbonyldiimidazole in dry tetrahydrofuran (THF) for 5 h. The benzoxazolinone derivatives thus formed were reacted with ethylbromoacetate in dry acetone containing K_2_CO_3_, leading to the formation of acetic acid ethyl ester derivatives which on further reaction with hydrazine hydrate in absolute ethanol yielded hydrazides. The formation of the ester derivatives was confirmed from the TLC and ^1^H NMR spectra which showed the presence of a singlet for two methylene protons in a range of δ 4.87–4.92. The formation of the ester derivatives was further confirmed by the appearance of two signals at δ 1.21 (t, *J* = 6.9 Hz, 3H) and 4.18 (q, *J* = 7.2 Hz, 2H) whereas the formation of the acetic acid hydrazide derivatives from the ester derivatives was confirmed by the disappearance of these two signals and the appearance of two new signals in the ^1^H NMR spectra at δ 9.1 (s, 1H, CONH) and δ 4.40 (s, 2H, NH_2_). The hydrazide derivatives were converted into corresponding thiosemicarbazides by reaction with different substituted aryl isothiocyanates in absolute ethanol. The presence of the extra signals in the aromatic region due the protons of the phenyl ring (δ 7.53–7.73) of the isothiocyanate along with the signals at δ 9.5–9.9 (CSNH) and 10.13 –10.58 (CONH) confirmed the formation of the thiosemicarbazides. The thiosemicarbazides were finally cyclised into respective substituted 1,3,4-thiadiazoles in the presence of sulphuric acid followed by neutralisation with liquid ammonia. All the synthesized compounds (Fig. 2) have been evaluated for their *in vivo* anti-inflammatory activity. The compounds showing significant *in vivo* anti-inflammatory activity were further evaluated for their *in vivo* antinociceptive activity and *in vitro* TNF-α inhibition along with their gastric risk evaluation.

### 3.2 *In vivo* anti-inflammatory activity

**Fig. 3:**
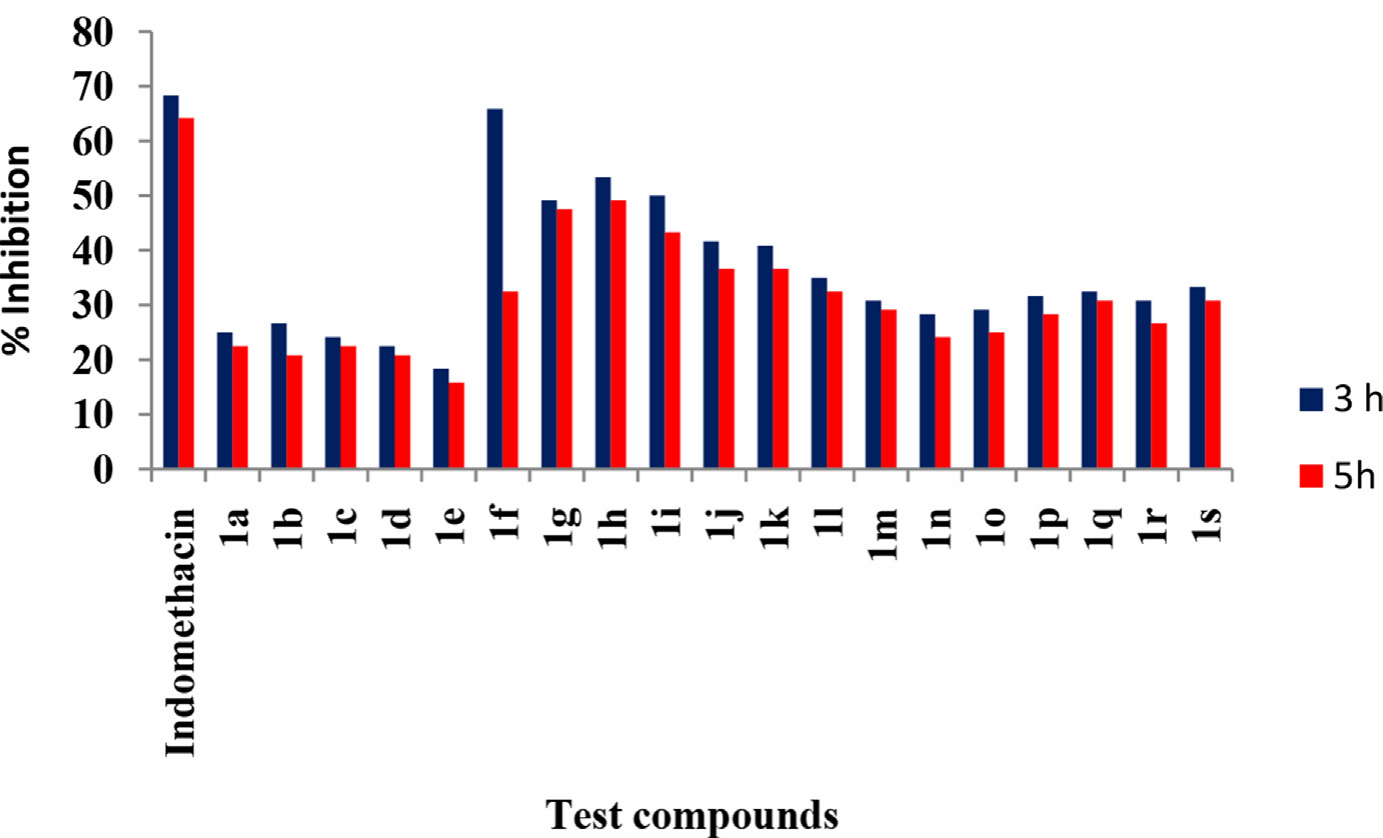
Results of the *in vivo* anti-inflammatory activity.

All the synthesized compounds were evaluated for their *in vivo* anti-inflammatory activity (Fig. 3) by carrageenan induced hind paw edema model. All the compounds showed a time-dependent decrease in the inhibition of inflammation after 3 h and 5 h. The compound **1f** exhibited a potent anti-inflammatory activity with an inhibition of 65.83% and 32.50% in comparison to indomethacin which showed 68.33% and 64.16% inhibition after 3 h and 5 h respectively. The compounds **1g - 1j** exhibited significant anti-inflammatory activity (p < 0.001) with an inhibition of 49.16%, 53.33%, 50.00% and 41.66% after 3 h and 47.50%, 49.16%, 43.33% and 36.66% respectively after 5 h.

The structure activity relationship has been developed on the basis of the nature and position of the substituents on the benzoxazolinone ring and the aryl ring attached to the 1,3,4-thiadiazole nucleus through the –NH linkage. The following order of the decreasing activity was observed with respect to the position of the substituents on the benzoxazolinone ring- **6CH**_**3**_ **> 5CH**_**3**_ **> 5Cl > H > 6NO**_**2**_^.^ The compounds containing electron-withdrawing nitro group at the sixth position exhibited the least activity whereas those containing electron-donating methyl group at the same position exhibited the most potent activity i.e. the compounds can be arranged in the following decreasing order of activity-

**1j** > **1a**, **1f** > **1b**, **1k** > **1e**, **1g** > **1c**

### 3.3 *In vitro* TNF- α assay

TNF-α plays an important role in the perpetuation of chronic inflammation and tissue damage during the development of inflammatory disorder. The compounds showing significant *in-vivo* anti-inflammatory activity were further screened for their *in-vitro* TNF-α activity (Table 1). The compound **1f** showed significant (p < 0.01), TNF- α inhibitory activity with 51.44 % inhibition as compared to the standard drug indomethacin which exhibited 62.05 % (p < 0.01) inhibition.

**Table 1:** *In vitro* TNF-α inhibition of the 1,3,4-thiadiazole-based benzoxazolinones.

Test Compound	Concentration (μg/ml)	Mean ± SEM	% Inhibition
LPS Control	1	3.11 ± 0.10	-
Indomethacin	1	1.18 ± 0.08**	62.05
1f	1	1.51 ± 0.15**	51.44
1g	1	1.70 ± 0.08**	45.33
1h	1	2.65 ± 0.08*	14.79
1i	1	2.66 ± 0.08*	14.46
1j	1	2.63 ± 0.09**	15.43

Data is analysed by one way ANOVA followed by Dunnett's ‘t’ test and expressed as mean ± SEM from six observations; ***p < 0.001, **p < 0.01 & *p < 0.05.

### 3.4 *In vivo* antinociceptive activity

**Fig. 4:**
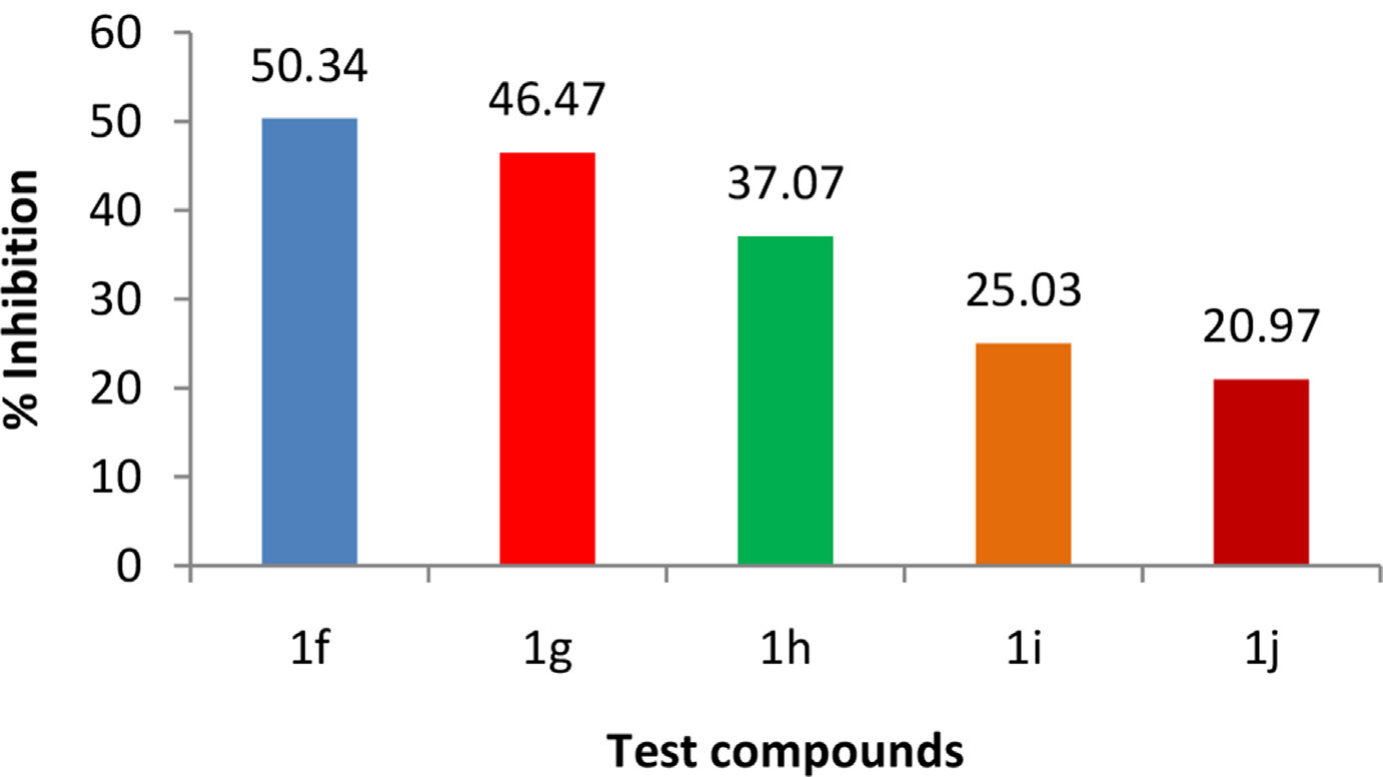
Results of the *in vivo* antinociceptive activity by writhing test.

The compounds showing significant *in vivo* anti-inflammatory activity in comparison to the standard drug indomethacin were further tested for their *in vivo* antinociceptive activity by the writhing test and tail immersion method. The results of the writhing test (Fig. 4) indicate that the compounds **1f, 1g, 1h, 1i** and **1j** exhibited potent antinociceptive activity with 50.34, 46.47, 37.07, 25.03 and 20.97% inhibition as compared to the standard drug indomethacin which caused 57.13% inhibition. The results of the tail immersion method (Table 2) show that the compounds **1g** and **1i** (p < 0.01) exhibited potent activity in comparison to indomethacin (p < 0.001).

**Table 2:** *In vivo* antinociceptive activity by the tail immersion method.

Group	Dosage	Basal Reaction time (min)	Reaction time (min)		
			30	60	120
Control	2 ml/kg	2.34 ± 0.14	2.44 ± 0.15	2.48 ± 0.16	2.52 ± 0.14
Indomethacin	0.05 mol/Kg	2.42 ± 0.06	3.56 ± 0.09^∗∗∗^	3.60 ± 0.07^∗∗∗^	3.66 ± 0.05^∗∗∗^
1f	0.05 mol/Kg	2.64 ± 0.05	2.42 ± 0.03	2.52 ± 0.03	2.58 ± 0.03
1g	0.05 mol/Kg	2.78 ± 0.05	2.96 ± 0.05^∗∗^	3.00 ± 0.03^∗∗^	3.04 ± 0.05^∗∗^
1h	0.05 mol/Kg	2.28 ± 0.08	2.90 ± 0.07^∗^	2.92 ± 0.04^∗^	2.94 ± 0.06^∗^
1i	0.05 mol/Kg	2.60 ± 0.07	2.98 ± 0.08^∗∗^	3.02 ± 0.05^∗∗^	2.96 ± 0.07^∗∗^
1j	0.05 mol/Kg	2.44 ± 0.05	2.58 ± 0.06	2.64 ± 0.09	2.70 ± 0.08

Data is analyzed by one way ANOVA followed by Dunnett's ‘t’ test and expressed as mean ± SEM from six observations; ***p < 0.001, ** indicates P < 0.01 & * indicates P < 0.05.

**Table 3:** Docking scores of reference ligand and 19 ligands with TNF-α protein.

Ligands	Glide Score	Activity
2AZ5 Ligand	-7.1	-48.92
1a	-5.48	-42.03
1b	-4.99	-40.22
1c	-5.39	-39.86
1d	-5.16	-41.89
1e	-5.20	-34.34
1f	-5.06	-35.29
1g	-4.97	-37.66
1h	-5.72	-40.74
1i	-5.77	-40.01
1j	-5.78	-40.13
1k	-5.80	-39.58
1l	-5.69	-39.80
1m	-5.62	-41.21
1n	-5.46	-40.56
1o	-5.52	-40.43
1p	-5.55	-37.88
1q	-7.04	-42.06
1r	-5.60	-40.84
1s	-5.50	-37.02

ADME (absorption, distribution, metabolism and excretion) predictions for the test molecules were made using the QikProp program. The van der Waals surface area of polar nitrogen and oxygen atoms (PSA), partition coefficient (log P o/w), and aqueous solubility (log S) are shown in Table 4. It can be concluded that the values of the calculated properties are within the standard range.

**Table 4:** *In silico* ADME properties of the test compounds with respect to TNF-α protein.

Ligands	Molecular Weight	Log S	PSA	LogP o/w
Indomethacin	357.78	-3.007	36.369	5.4
1a	403.01	-5.134	48.054	4.149
1b	399.06	-4.153	49.235	4.064
1c	387.04	-4.672	50.185	4.055
1d	446.96	-4.316	52.625	4.247
1e	369.05	-5.126	46.253	4.338
1f	368.09	-4.754	85.253	2.846
1g	372.04	-5.256	45.165	4.27
1h	382.11	-4.284	53.127	3.657
1i	356.07	-3.473	55.711	3.797
1j	372.04	-5.236	50.845	3.889
1k	338.08	-4.354	62.456	2.794
1l	356.07	-3.663	59.474	3.452
1m	415.99	-4.527	52.148	3.876
1n	401.97	-4.415	52.262	3.436
1o	358.02	-5.271	50.912	4.475
1p	342.05	-4.402	80.132	2.678
1q	369.05	-5.613	53.452	5.103
1r	391.99	-4.236	46.473	4.217
1s	376.01	-4.143	48.537	3.972

### 3.5 Gastric ulceration study

**Fig. 5:**
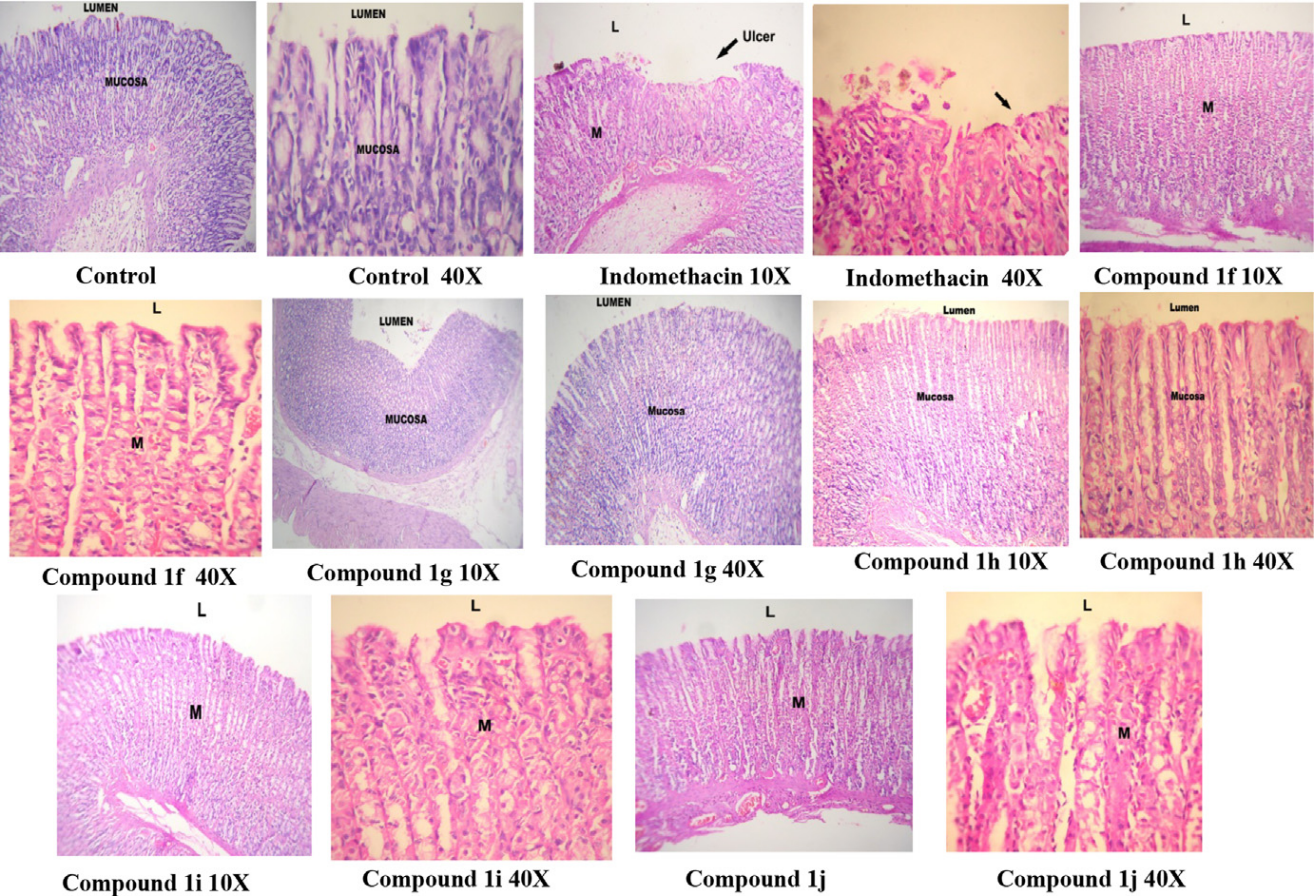
Histopathology report after ulcer induction in rats.

The compounds showing potential *in vivo* anti-inflammatory and *in vivo* antinociceptive activities were further tested for their gastric ulceration activity (Fig. 5). When compared with indomethacin, the compounds **1f, 1g, 1h, 1i** and **1j** did not cause any gastric ulceration and rupture of the gastric mucosal layer.

### 3.6 Molecular docking studies against TNF-α

**Fig. 6:**
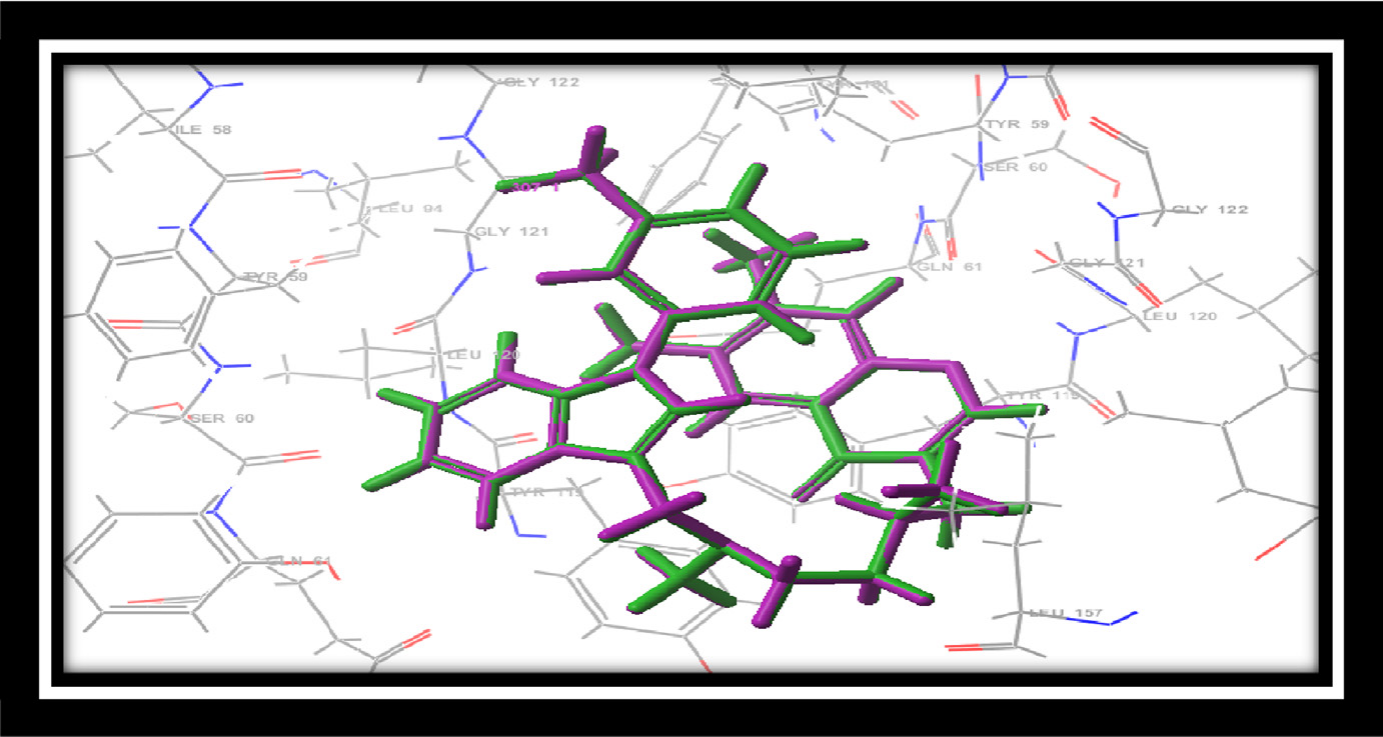
Superimposed binding orientation of the reference ligand (green) and docked reference ligand (maroon) as predicted by Schrodinger glide software.

The synthesized ligands have been subjected to the *in silico* molecular docking study against the TNF-α protein by using the 2AZ5 ligand which was downloaded from the protein data bank (PDB). The 2AZ5 ligand has a large binding pocket and its binding site is mainly hydrophobic containing leucine, glycine, and tyrosine amino acid residues. The ligands should also be hydrophobic in nature in order to bind with the large hydrophobic pocket of 2AZ5. The reference ligand was docked separately with the generated grid to validate the grid and docking methodology. Fig. 6 shows comparison between the original binding mode of reference ligand as predicted by Schrodinger glide software. It can be clearly seen that adapted Schrodinger methodology successfully predicted the binding of crystallographic 2AZ5 ligand with root mean square deviation of 0.003^0^A.

**Fig. 7:**
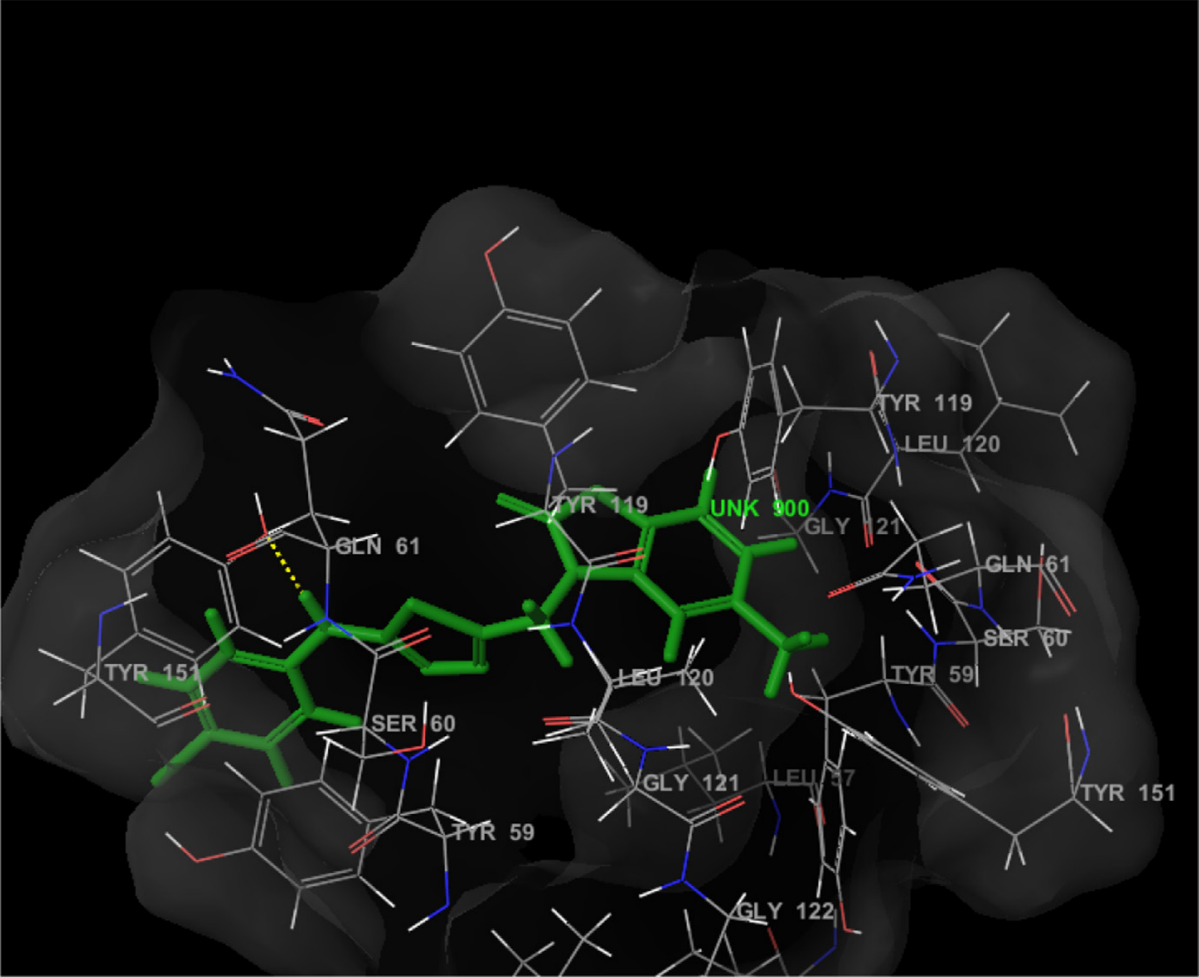
The binding interactions of compound **1f** with TNF-α protein.

The compound **1f** was found to fit in the binding pocket (Fig. 7) of TNF-α protein and showed hydrogen bonding with GLN 61 and binding interactions with TYR 119, TYR 151 and GLY 121. Its glide score was found to be **-5.06**. The Table 3 shows the glide scores of the synthesized compounds against the TNF-α protein.

## 4 Conclusion

We have synthesized a focussed library of 1,3,4-thiadiazole-based benzoxazolinones containing benzoxazolinone and 1,3,4-thiadiazole nuclei connected through a methylene linkage and evaluated them for their *in vivo* anti-inflammatory and antinociceptive activities. Pharmacological evaluations have revealed that the compounds containing 2-benzoxazolinone nucleus exhibit analgesic, anti-inflammatory, antineoplastic, anticonvulsant and antimicrobial activities. Zoxazolamine, a benzoxazole analogue, has been used as a skeletal muscle relaxant. Food and Drug Administration (FDA) has approved the usage of Lorzone tablet (Chlorzoxazone) as a muscle relaxant and it contains 5-chloro-2-benzoxazolinone as the key constituent. However the exact mode of action of 2-benzoxazolinone containing molecules is still a subject of further exploration. The structural diversity generated on 2-benzoxazolinone nucleus would help in understanding their mode of action in future. New structural modifications of the 2-benzoxazolinone core would result in the discovery of new target based molecules. The compounds **1f**, **1g**, **1h**, **1i** and **1j** exhibited potent anti-inflammatory and antinociceptive activities. The *in vitro* TNF-α assay shows that the compounds **1f** and **1g** exhibit significant TNF-α inhibitory activity as compared with indomethacin. The compounds **1f**, **1g**, **1h**, **1i** and **1j** exhibiting potent *in vivo* anti-inflammatory did not cause any gastric ulceration as compared to indomethacin. The *in silico* molecular docking study against TNF-a protein shows that the molecule **1f** shows interactions with TYR 119, TYR 151.

## Declarations

### Author contribution statement

Saqlain Haider: Performed the experiments; Wrote the paper.

Mohammad S. Alam: Conceived and designed the experiments.

Hinna Hamid, Abhijeet Dhulap: Analyzed and interpreted the data.

Deepak Kumar: Contributed reagents, materials, analysis tools or data.

### Funding statement

This research did not receive any specific grant from funding agencies in the public, commercial, or not-for-profit sectors.

### Competing interest statement

The authors declare no conflict of interest.

### Additional information

No additional information is available for this paper.
